# Supplementary material for: Ion-Channel-Targeting Drugs for Chikungunya Virus
Source: Molecules. 2025 Oct 1;30(19):3942. doi: 10.3390/molecules30193942 (PMC12525687; doi:10.3390/molecules30193942)
Supplement: Supplementary file 1 [file molecules-30-03942-s001.zip › molecules-3857447-supplementary.pdf]

Ion Channel Targeting Drugs for Chikungunya Virus

Hiya Lahiri[1,3,*], **Kingshuk Basu**[1,3,*], **and Isaiah T. Arkin**[1,2,*]

[1]Department of Biological Chemistry, The Alexander Silberman Institute of Life Sciences, The Hebrew University of Jerusalem, Edmond J. Safra Campus, Jerusalem, 9190400, Israel.
[2]Department of Biomedical Engineering, City University of Hong Kong, Kowloon, Hong Kong, China.
[3]Current address: Department of Biomedical Engineering, City University of Hong Kong, Kowloon, Hong Kong, China.
[*]hiya.lahiri@mail.huji.ac.il
[*]kingshuk.baso@mail.huji.ac.il
[*]Isaiah.Arkin@cityu.edu.hk

ABSTRACT

Alphaviruses are transmitted by Aedes mosquitoes and cause large-scale epidemics worldwide. Chikungunya virus (CHIKV) infection can cause febrile seizures known as chikungunya fever (CHIKF), which ultimately leads to severe joint pain and myalgia. While a vaccine has recently been introduced against CHIKV, at present, no anti-viral drug is available. CHIKV, like other alphaviruses, has a short 6K protein capable of forming an ion channel. Blocking this ion channel with drugs can therefore serve as a potential way to curtail CHIKV infection. To that end, we screened a repurposed drug library using three bacteria-based channel assays to detect blockers against 6K viroporin, yielding several hits. Interestingly, several of the blockers were able to inhibit the 6K protein from the similar Eastern equine encephalitis virus (EEEV), while others were not, pointing to structural specificity which may be explained by modeling studies. In conclusion, our study provides a starting point for developing a new route to potentially inhibit CHIKV.

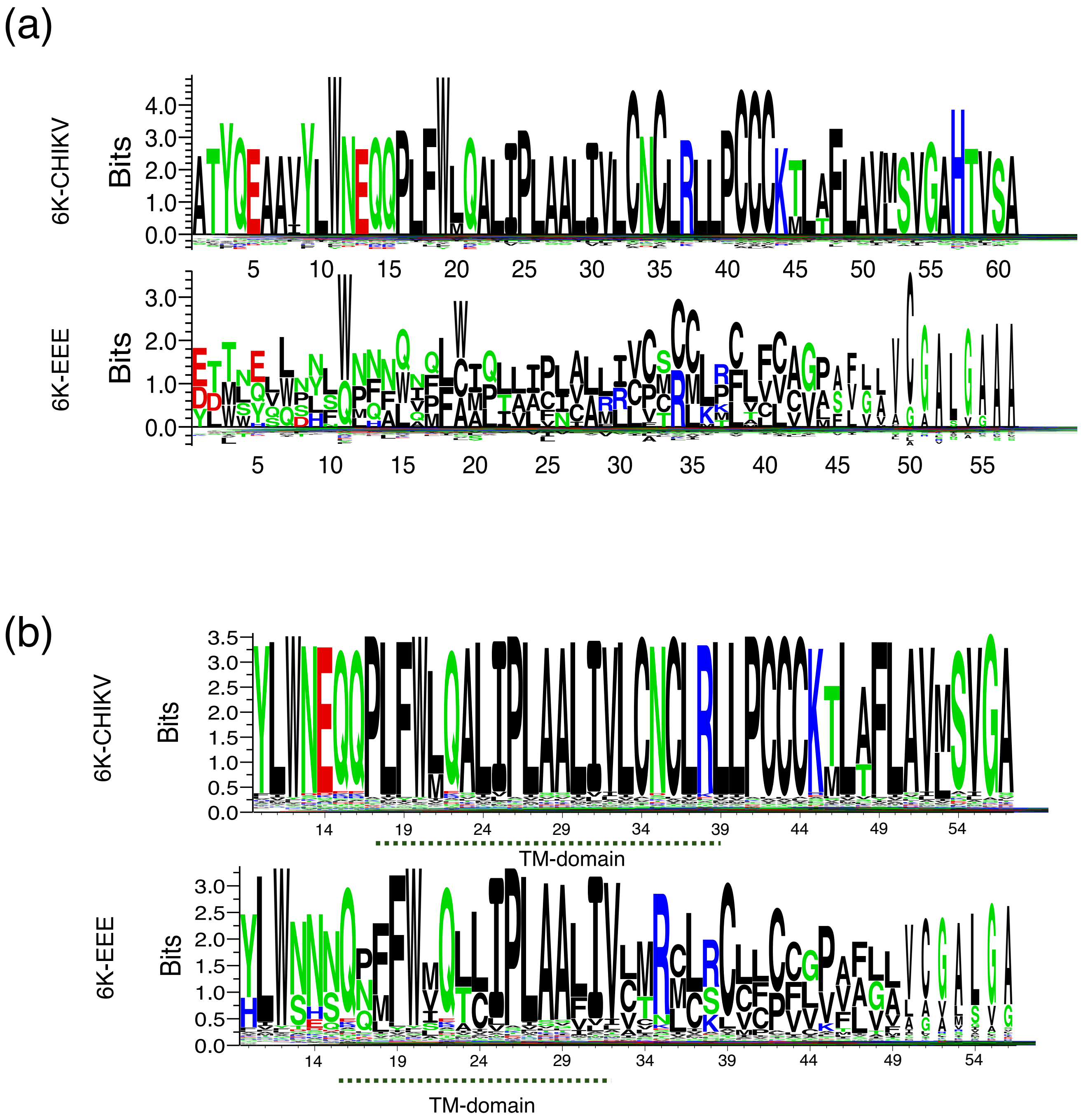

Figure S1. (a) Sequence conservation of 6k proteins from Chikungunya virus and Eastern equine encephalitis virus represented as logo images for each protein. The height of the letters denoting one letter code of an amino acid represents the statistical prevalence of that particular amino acid in that position. (b) Aligned sequences of 6K-Chikungunya virus and 6K-Eastern equine encephalitis virus showing aligned sequences from 9 to 56.

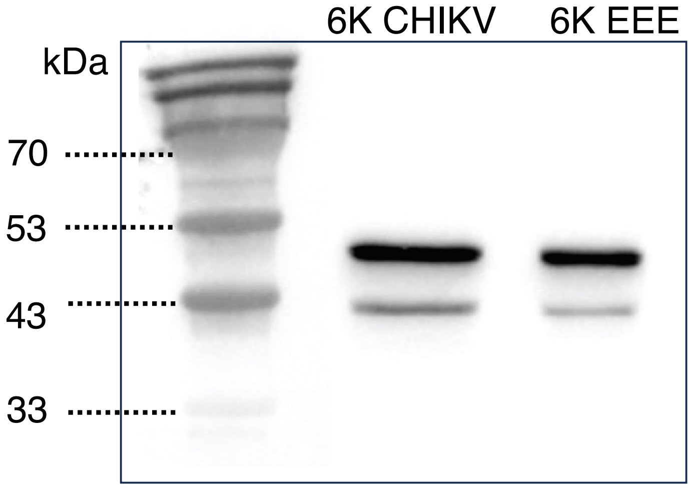

Figure S2. Western blot of the 6K CHIKV and 6K EEEV chimeric constructs expressed in DH10B cells at 0.1 mM IPTG concentration. The presence of a band for both proteins at around 50 kDa confirms the protein expression.

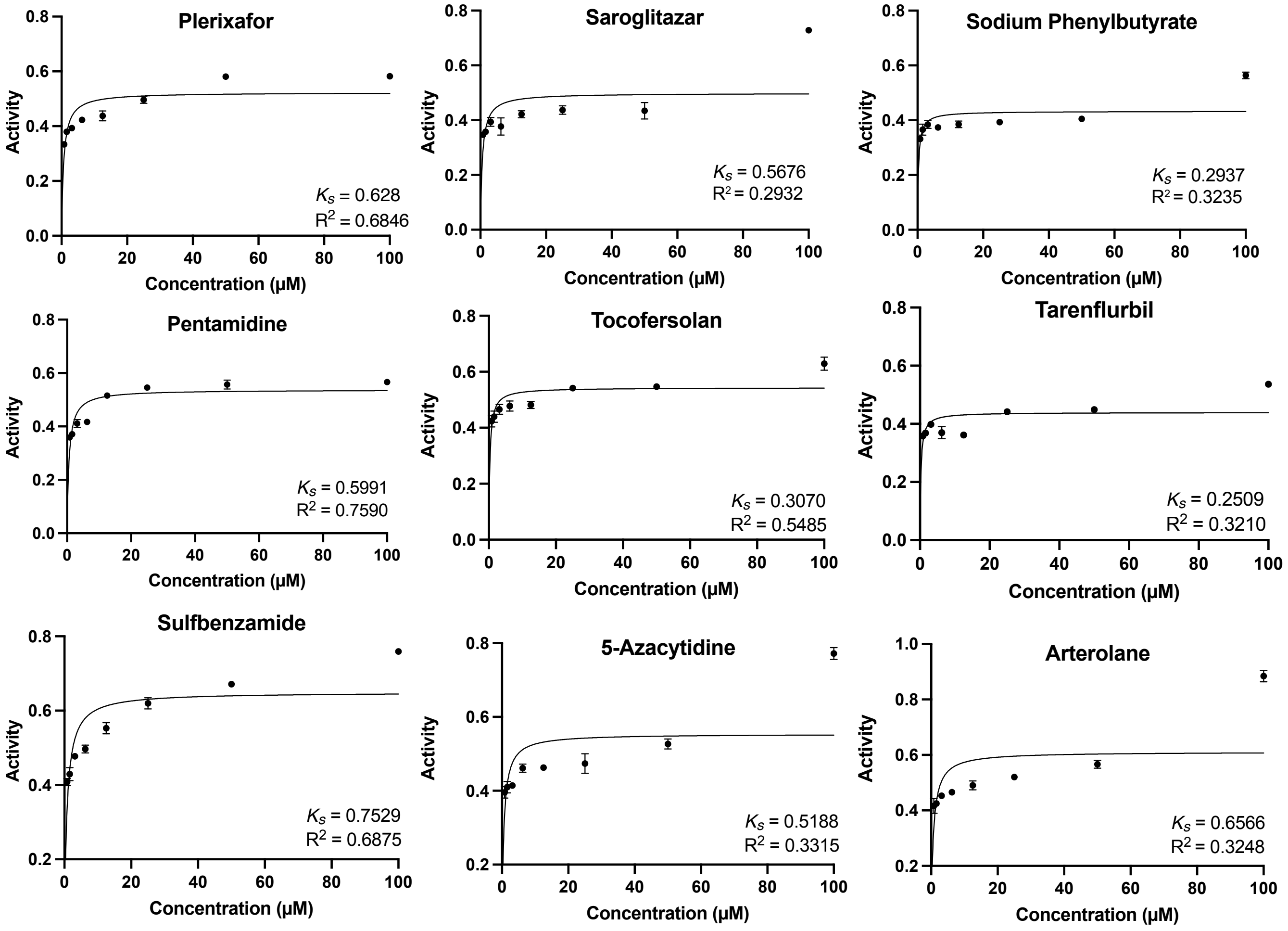

Figure S3. Monod coefficients (K_s) for each blocker identified in the bacteria-based assays shown in Figure 3.

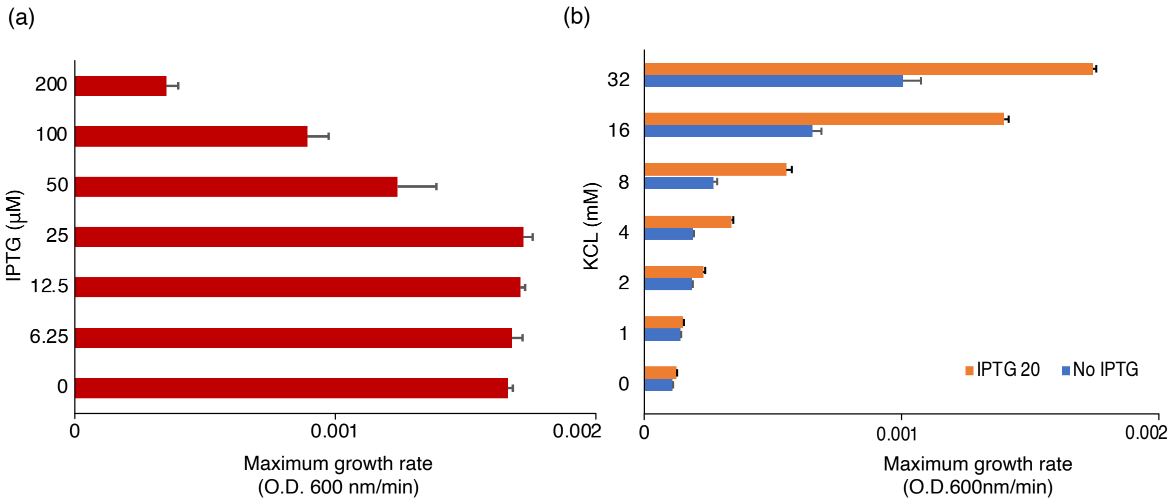

Figure S4. Bacteria based channel assays to assess the ion channel activity of 6K EEEV viroporin (a) negative assay and (b) positive assay.

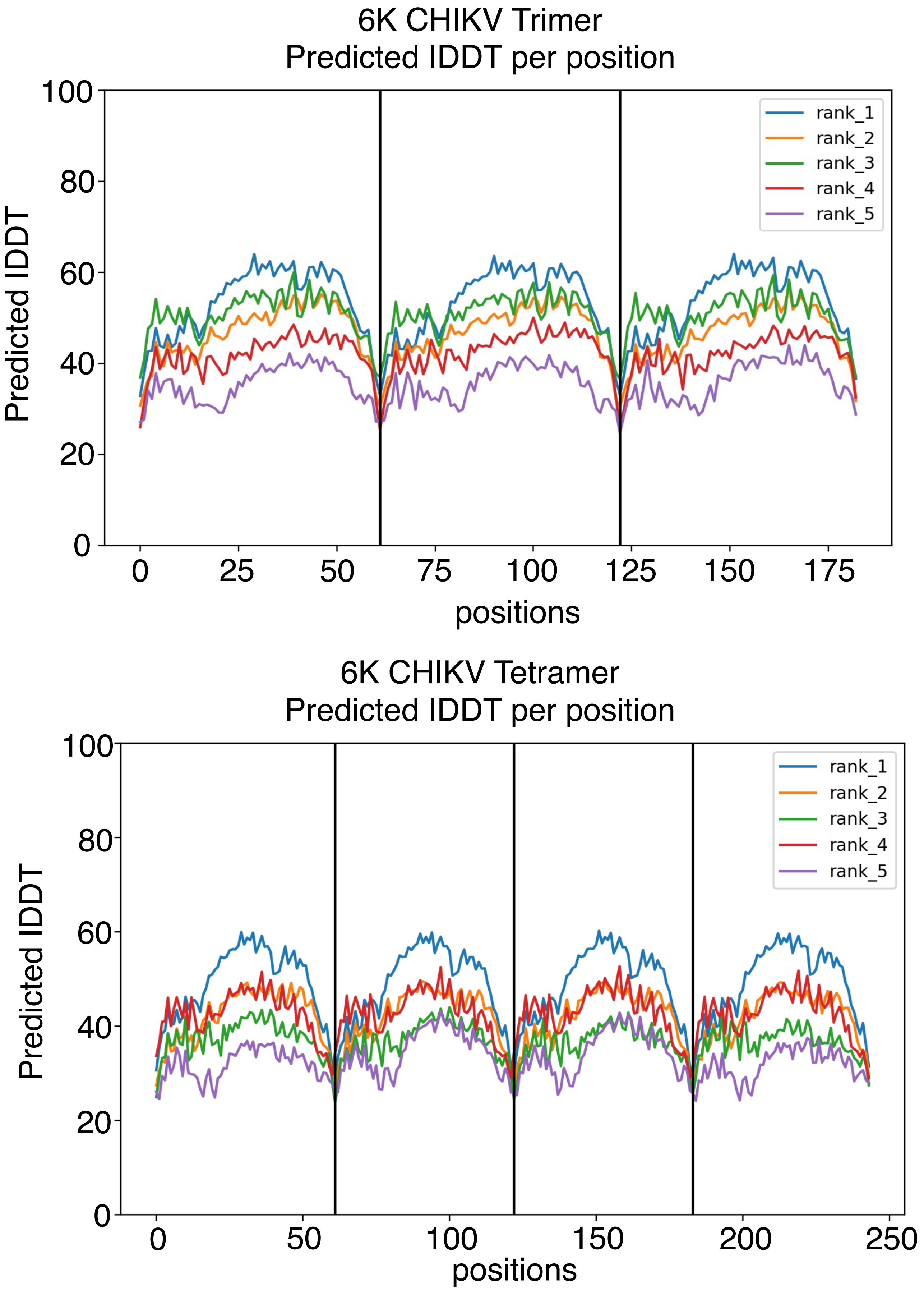

Figure S5. PLDDT vs position plot for trimer and tetramer of 6K CHIKV, obtained from AlfaFold2.

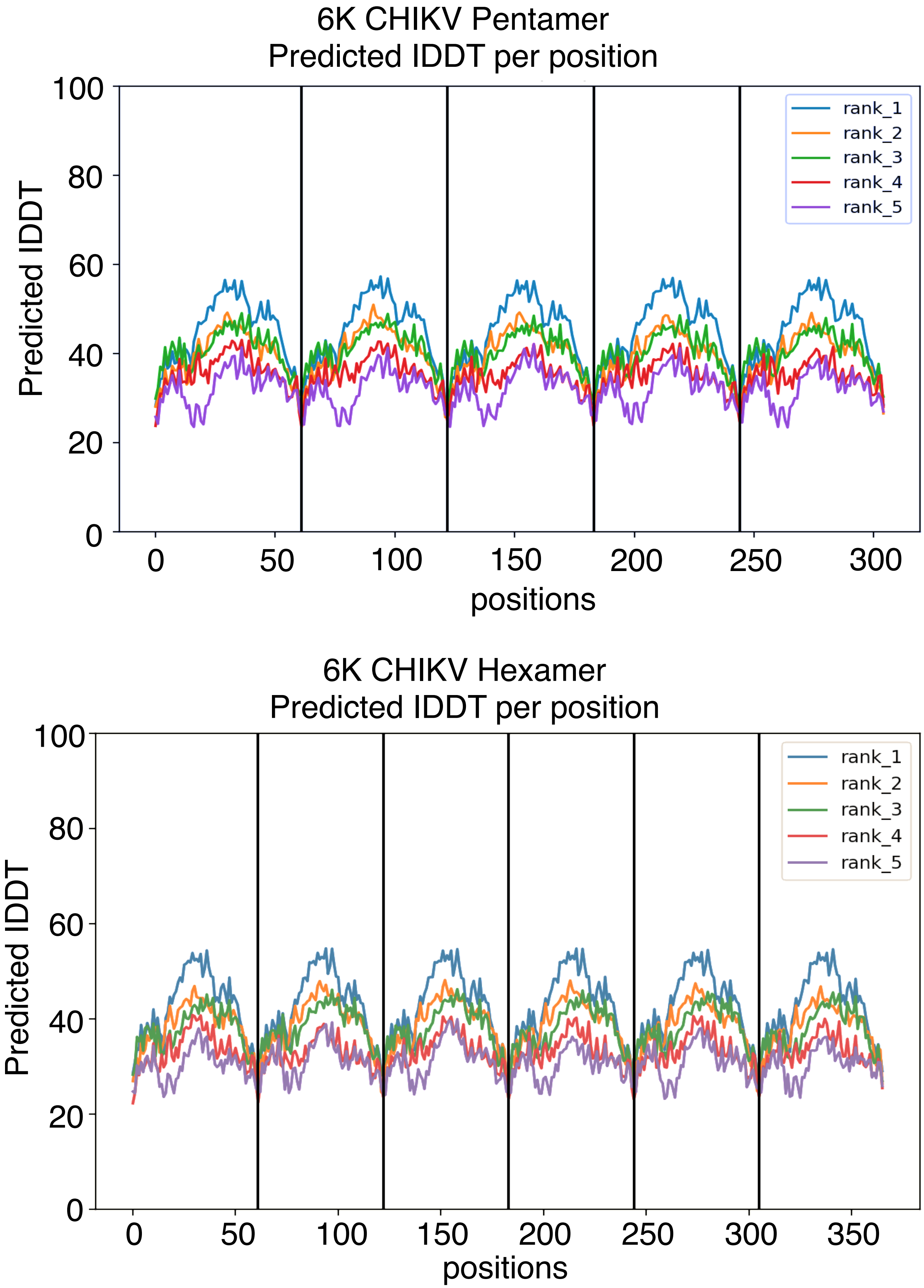

Figure S6. PLDDT vs position plot for pentamer and hexamer of 6K CHIKV, obtained from AlfaFold2.

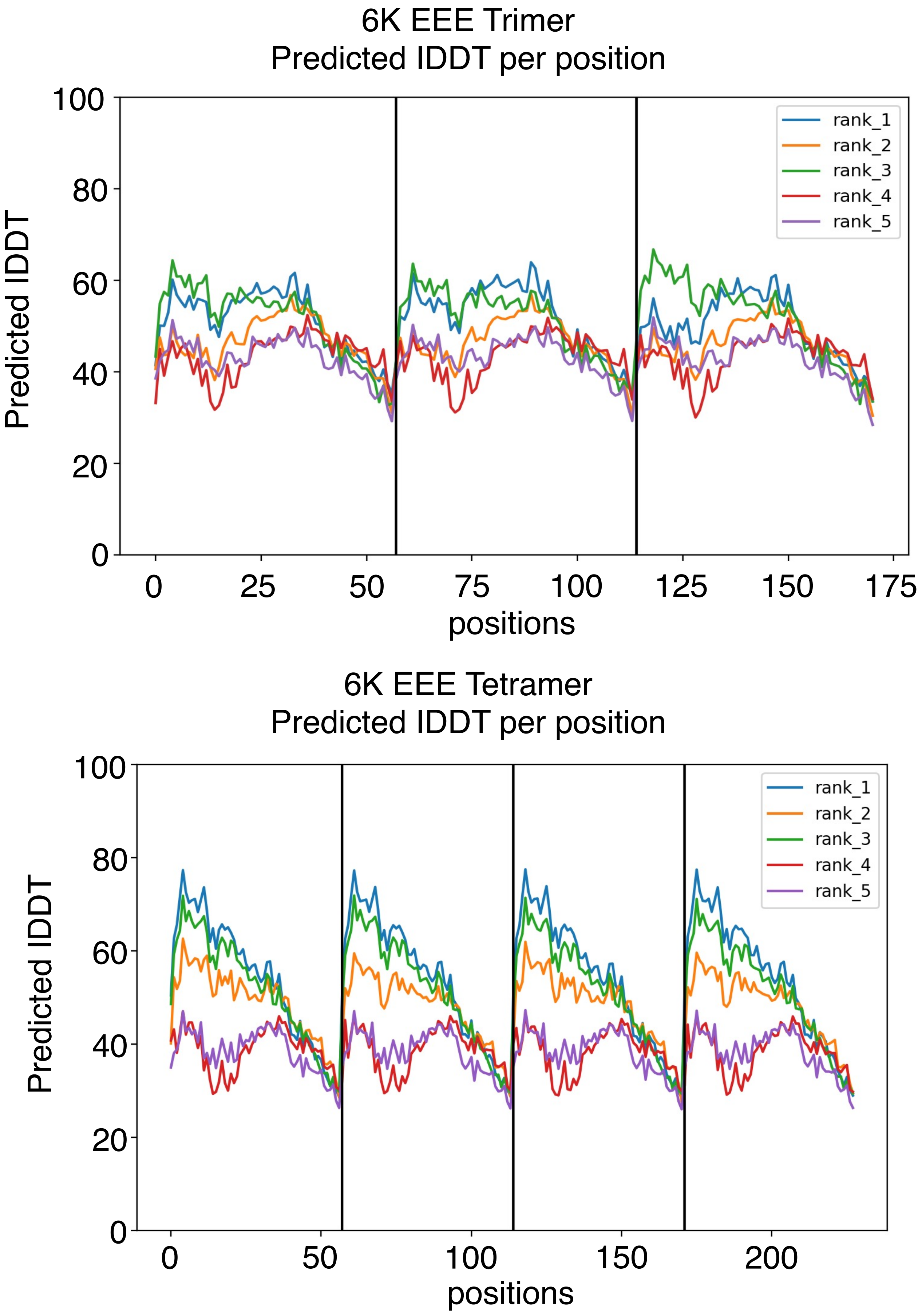

Figure S7. PLDDT vs position plot for trimer and tetramer of 6K EEE, obtained from AlfaFold2.

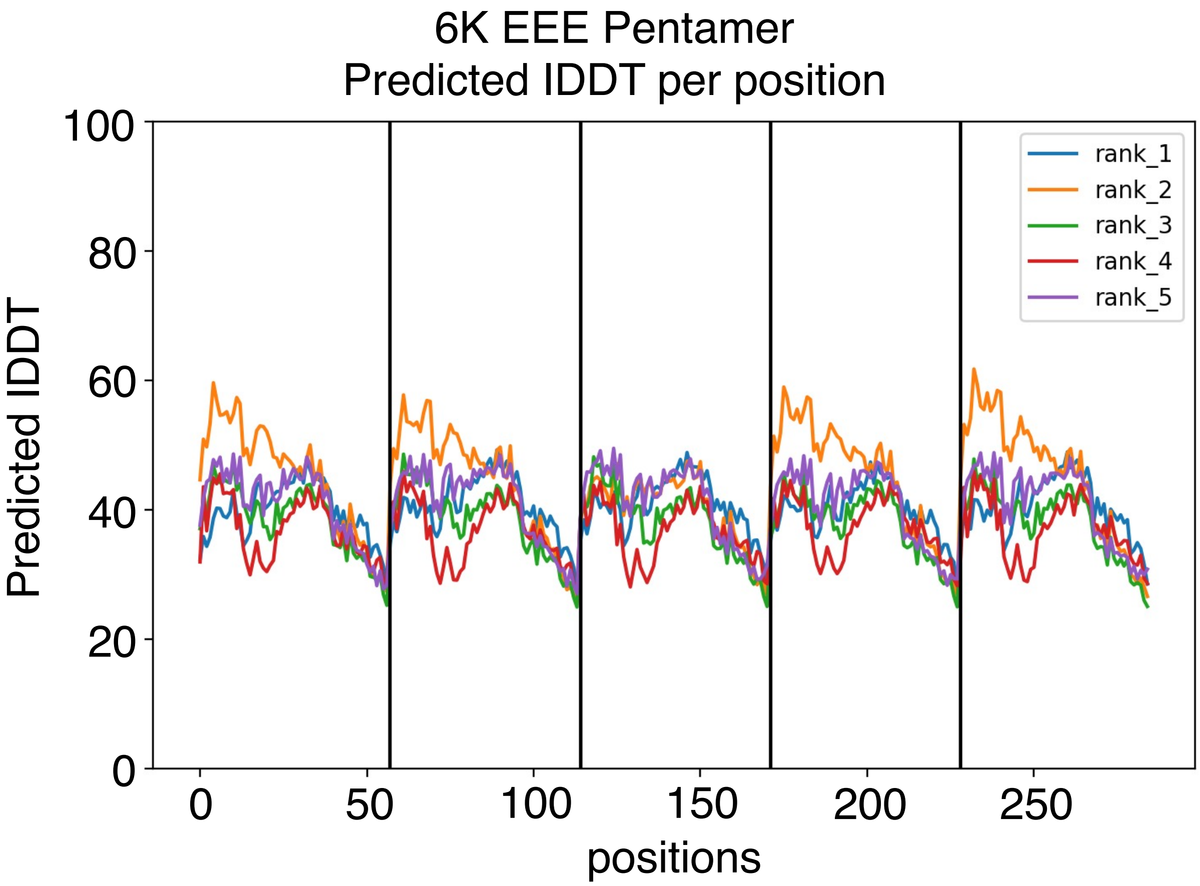

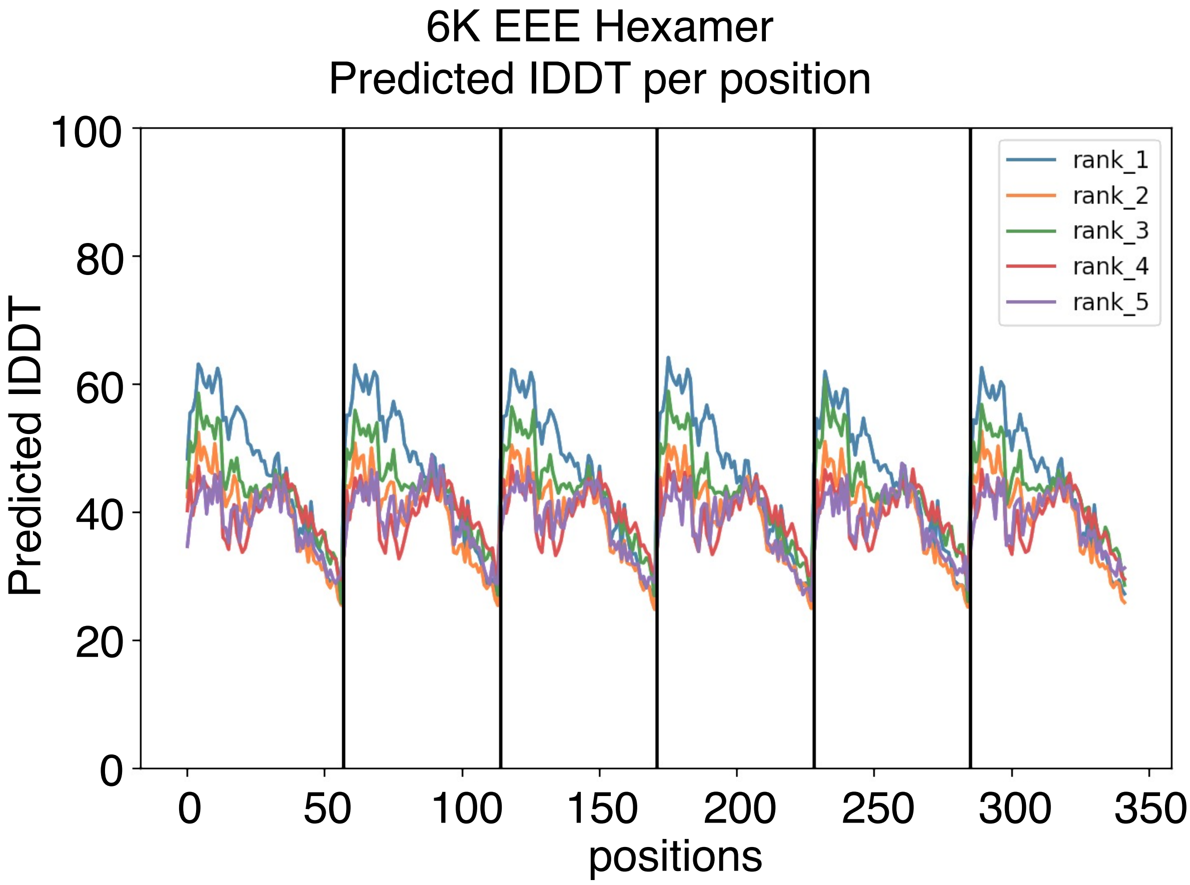

Figure S8. PLDDT vs position plot for pentamer and hexamer of 6K EEE, obtained from AlfaFold2.

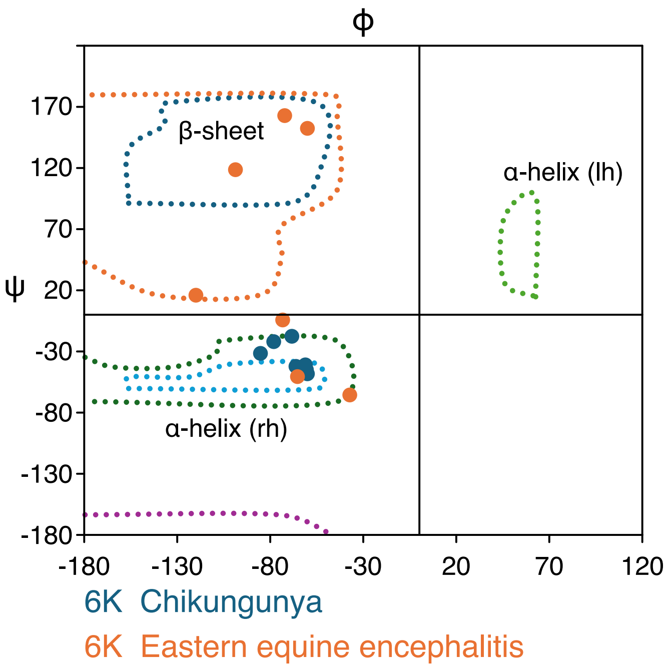

Figure S9. *phi-psi* plot of post-transmembrane region (from residue 40 to 46) of 6K CHIKV (blue) and 6K EEE (orange) averaged over trajectory of MD simulation (from 100 ns to 200 ns)

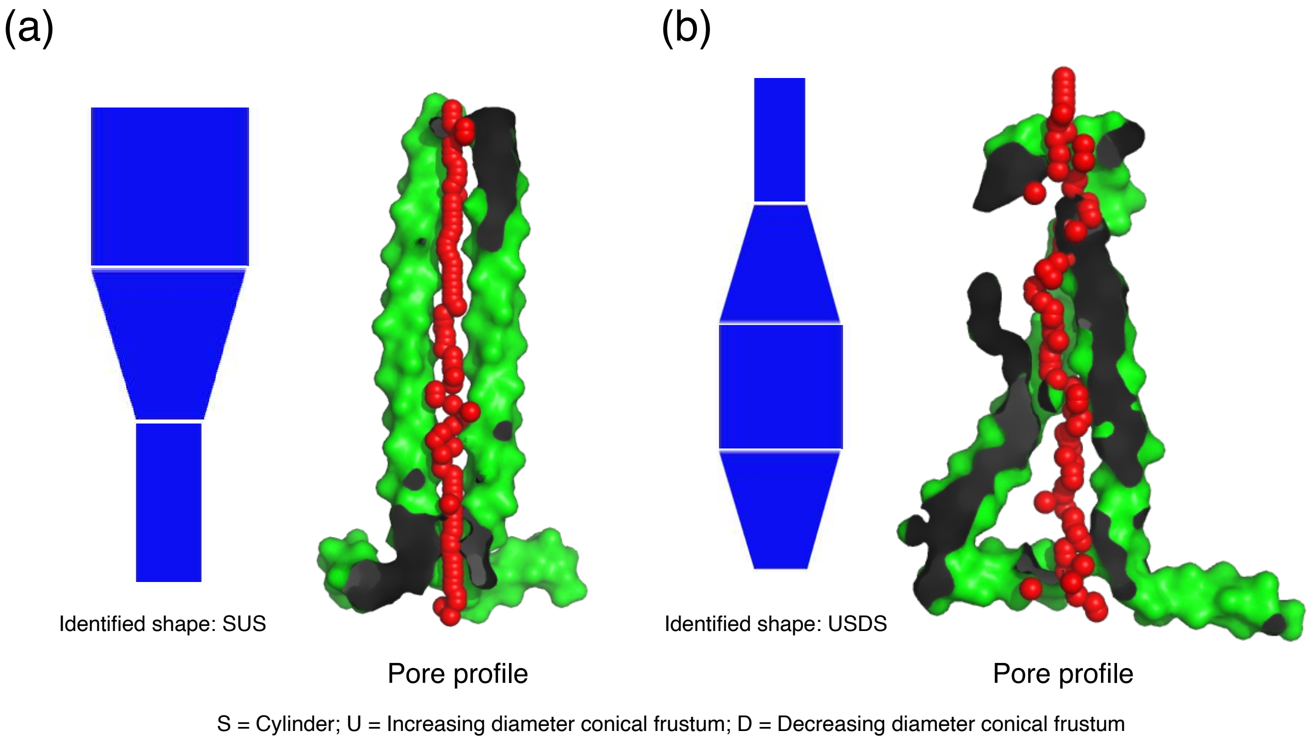

Figure S10. Pore shape analysis of 6K (a) CHIKV and (b) EEE using PoreWalker software.
